# Exploring Motor–Cognitive Interference Effects and the Influence of Self-Reported Physical Activity on Dual-Task Walking in Parkinson’s Disease and Healthy Older Adults

**DOI:** 10.3390/brainsci15020114

**Published:** 2025-01-25

**Authors:** Thomas Jürgen Klotzbier, Nadja Schott, Soo-Yong Park, Quincy J. Almeida

**Affiliations:** 1 Institute of Sport and Movement Science, University of Stuttgart, 70569 Stuttgart, Germany; nadja.schott@inspo.uni-stuttgart.de (N.S.); soo-yong.park@inspo.uni-stuttgart.de (S.-Y.P.); 2 Parkinson’s & Neurodegenerative Disease Programs, Carespace Health & Wellness, Waterloo, ON N2L 3C5, Canada; qalmeida@carespace.health

**Keywords:** dual-task interference, Parkinson’s disease, motor subtypes, motor–cognitive interaction, physical activity, gait and balance, cognitive load

## Abstract

**Introduction:** Parkinson’s disease (PD) is characterized by motor and cognitive impairments that often manifest as distinct motor subtypes: Postural Instability Gait Difficulty (PIGD) and Tremor-Dominant (TD). Motor–cognitive interference, especially under dual-task (DT) walking conditions, may vary by subtype, providing insights into specific impairments. This study explored DT interference effects in PD subtypes, focusing on the potential impact of self-reported physical activity, which may help mitigate subtype-specific impairments and improve motor–cognitive function. **Methods:** PD patients classified as PIGD or TD and healthy controls completed single-task (ST) and DT walking assessments involving different cognitive tasks (Serial Subtraction, Auditory Stroop, and Clock Task). Physical activity levels were evaluated using the CHAMPS questionnaire, analyzing the self-reported frequency and duration of weekly exercise-related activities. **Results:** Interference effects were significantly different between PD patients and controls, with the PIGD group showing greater motor impairment under high cognitive load, primarily affecting gait, than the TD and control groups. Performance differences between groups diminished as cognitive load increased. Self-reported physical activity does not significantly moderate motor performance under DT conditions, suggesting that activity levels in this sample are insufficient to offset motor–cognitive interference. However, like group affiliation, physical activity directly influences motor performance during DT conditions, indicating that both factors independently impact motor–cognitive function in PD. **Discussion:** These findings suggest that DT assessments help differentiate PD motor subtypes, as group differences were minimal in ST conditions. While physical activity is associated with general improvements in motor ST and DT performance in PD and controls, the lack of a significant moderating effect from self-reported exercise-related physical activity indicates that current activity levels may not be high enough to counter motor–cognitive interference. More intensive or DT-specific exercise may be required to reduce interference effects. Future research should examine the role of structured physical activity programs, potentially incorporating DT training, to evaluate their impact on motor–cognitive interference in PD.

## 1. Introduction

Parkinson’s disease (PD) is a neurodegenerative disorder characterized by a variety of motor symptoms, notably tremors, bradykinesia, rigidity, and postural instability. These symptoms significantly affect the mobility of affected individuals and often result in motor impairments, particularly during walking [1]. It has been demonstrated that within the Parkinson’s population, there are differences in motor symptoms leading to various motor subtypes [2,3,4,5,6]. Particularly noteworthy are the PIGD (Postural Instability and Gait Difficulty) subtype, characterized by postural instability and gait problems, and the TD (Tremor-Dominant) subtype, characterized by predominant tremor [7].

Investigating interference effects under single-task (ST) and dual-task (DT) conditions in subjects with PD can provide valuable insights into the severity of motor impairments [8]. This approach can clarify how PD affects both motor and cognitive domains, particularly as motor subtypes (such as Tremor-Dominant and Postural Instability/Gait Difficulty subtypes) may display unique interaction patterns between cognitive tasks and motor performance [7,9]. Also, DT studies are crucial for understanding motor–cognitive interference effects because they simulate real-life scenarios where people must manage both motor and cognitive tasks simultaneously, such as walking while talking [10]. In PD, motor–cognitive interference is likely amplified due to basal ganglia dysfunction, which impairs both automaticity in motor tasks [11] and executive functions, including learning, attention, and working memory [12]. In recent years, research has increasingly focused on analyzing motor impairments during walking in PD patients to identify potential specific gait patterns that could enable early diagnosis and targeted therapies [13,14,15,16].

In this context, numerous clinical studies, systematic reviews, and meta-analyses have demonstrated the positive effects of physical activity and exercise on motor symptoms in PD [15,16,17] or shown a slowing of symptom progression [18]. Several cohort studies have indicated that individuals with low levels of physical activity are at a higher risk of developing PD [13]. In a large prospective cohort study, greater intensity of physical activity was associated with a greater reduction in PD risk [19,20]. Also, the long-term benefits of regular physical activity have been linked to a slower decline in postural control and gait stability, daily living activities, and processing speed in patients with early-stage PD [14]. While research suggests that chronic physical activity improves overall motor function and cognitive performance, there is a notable gap in studies explicitly addressing the impact of exercise-related physical activity on motor–cognitive interference effects. Most existing studies focus on the impact of DT training [21,22] rather than investigating how physical activity directly influences interference effects in DT situations.

The present study aims to capture and analyze the above-mentioned aspects in a population-based investigation. Healthy older adults will be utilized as a control group to determine specific differences between healthy individuals and those with PD. Additionally, the motor subtypes PIGD and TD within the PD population will be separately examined to identify potential divergent motor–cognitive interferences. The central question of this study was to evaluate the extent to which interference patterns differ between healthy subjects and those with PD and whether exercise-related physical activity influences motor performance under DT conditions. Based on existing knowledge and hypotheses, we anticipated three main outcomes: First, we expected significant motor and cognitive performance differences between healthy subjects and PD. Second, we expected differences in interference patterns between motor subtypes within the groups. Third, we expected direct and indirect effects of exercise-related physical activity on motor DT performance.

## 2. Methods

### 2.1. Sample Characteristics

Participants were recruited from the Sun Life Financial Movement Disorders Research and Rehabilitation Centre database at Wilfrid Laurier University (MDRC; Waterloo, Ontario), comprising individuals diagnosed with idiopathic PD and age-matched healthy controls. Participants were recruited from a proprietary PD database where their PD status had been previously verified. All participants were required to read and sign an informed consent letter before participation. Data were collected from two distinct PD groups: one characterized by Postural Instability Gait Difficulty (PIGD) and another representing the Tremor-Dominant (TD) subtype. A third group of healthy control participants was also assessed using the experimental paradigm (see Table 1). Detailed inclusion and exclusion criteria are available in the Supplementary Materials.

### 2.2. Classification of Motor Subtypes in Parkinson’s Disease

The Unified Parkinson’s Disease Rating Scale (UPDRS; [23]) is structured into four key sections: (1) cognitive function, behavior, and mood; (2) activities of daily living (ADL); (3) motor function assessment, and (4) treatment-related complications. The motor section of the UPDRS-III specifically assesses the primary motor symptoms of PD, including tremors, rigidity, bradykinesia, and postural instability. Following the evaluation and using Jankovic et al.’s classification criteria (1991), this scale enables categorization into distinct PD motor phenotypes: the Tremor-Dominant type (TD), identified when the mean tremor score divided by the mean PIGD score is ≤1.5, and the Postural Instability and Gait Disorder type (PIGD), identified when the mean tremor score divided by the mean PIGD score is ≥1.0. Specific items within the scale were used to assess these two motor phenotype classifications (see Table 2 in [7]).

### 2.3. Assessment of Physical Activity Levels

The Activities Questionnaire for Older Adults (CHAMPS; [24]), a 41-item questionnaire, assesses the weekly frequency and duration of various physical activities that older adults may perform. Activity intensities range from light (e.g., leisurely walking and water exercises) to vigorous (e.g., walking or hiking uphill, jogging or running, and stationary biking). The questionnaire includes daily physical activities (e.g., gardening and housework) and recreational activities (e.g., golf and tennis). At the end of the questionnaire, participants can also add other activities. We calculated the total weekly time spent on exercise-related activities by multiplying the duration by the frequency for each listed exercise-related activity and then summing these values across all activities. For a detailed description of calculating the physical activity level based on exercise-related activities (see [24]).

### 2.4. Assessment of Motor and Cognitive Performance in Single and Dual Tasks Performance

#### 2.4.1. Single Task Conditions: Motor and Cognitive Performance

In the ST motor condition, participants walked a 10 m distance without any extra cognitive task. Upon reaching the 10 m mark, they were required to circle a cone and return along the same path. Participants began walking two meters before the start line, allowing for the acceleration and deceleration phases to be accounted for. Motor performance was assessed by the time taken to complete the 20 m walk, measured with a stopwatch.

Participants sat in front of a laptop for the ST cognitive condition, where visual stimuli were presented. The following three cognitive tasks were performed:

- Serial Subtraction Task (SS): Participants counted backward verbally from 100 in steps of three (e.g., “100–97–94”), with the experimenter recording errors and the total number of subtractions. Accuracy for the Subtraction Task was calculated as the number of correct subtractions divided by the total number of attempts, multiplied by 100 to yield the accuracy rate.
- Auditory Stroop Task (ST): Participants listened to the words “high” and “low” spoken in either a high (360 Hz) or low pitch (180 Hz). They indicated the pitch (not the word meaning) by verbally responding with “high” or “low” as quickly and accurately as possible. Stimuli were presented one second after the previous response. Missing responses were marked as errors and accuracy rates were calculated.
- Clock Task (CL): This visuospatial working memory task presented auditory prompts stating a time (e.g., “one-oh-five”). Participants mentally visualized this time on a clock face and responded by pressing a mouse button to indicate whether the clock hands were on the same side (“yes”) or opposite sides (“no”) of a vertical line bisecting the clock. Missed responses were marked as errors and accuracy rates were calculated.

#### 2.4.2. Dual Task Conditions: Combined Motor and Cognitive Performance

In the DT motor–cognitive conditions, participants walked the same 20 m walkway while simultaneously performing one of three cognitive tasks. Participants held a wireless mouse in their right hand to respond to tasks requiring button presses, while the experimenter manually recorded verbal responses for tasks without screen stimuli (Serial Subtraction).

### 2.5. Experimental Procedure

The participants were provided with detailed information about the objectives and content of the study, including the test procedures, duration, and any potential risks associated with data collection. Before participation, written informed consent was obtained. The methods employed in this study comply with the ethical principles outlined in the Declaration of Helsinki [25], national legislation, and applicable international norms and standards. The research project was granted ethical approval (REB #4791, Project: “Motor-Cognitive Interference in Dual Tasks: Allocation of Resources in Parkinson’s Disease Patients”, approved on 19 February 2016). Participant recruitment and data collection were conducted in March 2016.

Before experimental testing, participants underwent the Unified Parkinson’s Disease Rating Scale Part III (UPDRS-III) assessment to classify them into motor subtypes. PD participants completed the study while OFF their regular dopaminergic medication dosage, following a withdrawal period of 48 h for dopamine agonists and 12 h for levodopa and COMT inhibitors. Healthy control participants underwent the same experimental procedures, excluding the UPDRS-III assessment. The experimental protocol lasted approximately 120–150 min and comprised four trials: 1 ST and 3 DT walking trials. Each trial required participants to walk at their self-selected speed along a 10 m path, turn at the end of the first 10 m, and walk back, ensuring sufficient steady-state gait cycles for analysis. During ST and DT trials, participants completed three cognitive tasks for 60 s each. Accuracy was recorded using auditory stimuli presented through laptop speakers and E-Prime software 2.0. Experimental conditions were randomized to avoid any practice effects and prevent participants from developing strategies to perform the tasks. Throughout the study, a trained movement disorders specialist was present within the building to assist with any issues during data collection. All investigators were trained in First Aid and Cardiopulmonary Resuscitation (CPR), and they completed the Tri-Council Policy Statement: Ethical Conduct for Research Involving Humans Course on Research Ethics (TCPS 2: Core). Additionally, a spotter accompanied each participant during trials, ready to assist if any instability was detected. None of the participants experienced complications during data collection.

### 2.6. Statistical Analysis

All statistical analyses were conducted using SPSS version 30 (SPSS, Chicago, IL, USA). Group differences in continuous variables, such as age, height, weight, BMI, and physical activity, were examined using Analysis of Variance (ANOVA). To assess effect sizes, partial eta squared (*η*^2^_p_) values were reported, following Cohen’s (1988) guidelines for interpretation [26]: 0.01 for a small effect, 0.06 for a medium effect, and 0.14 for a large effect. Additionally, categorical demographic variables, including sex, were analyzed using the Chi-square (χ^2^) test to determine potential group differences.

A 3 (Group: PIGD, Tremor, Control) × 4 (Condition: ST_Walking, DT_SerialSubtraction, DT_Stroop, DT_Clock) repeated-measures ANCOVAs, controlling for weekly exercise-related physical activity (time/week spent in all exercise-related activities), were conducted to examine differences in ST and DT walking performance between healthy participants and Parkinson’s patients. A 3 (Group: PIGD, Tremor, Control) × 3 (Condition: Condition: ST_Walking, DT_SerialSubtraction, DT_Stroop, DT_Clock) repeated-measures ANCOVAs, controlling for weekly exercise-related physical activity (time/week spent in all exercise-related activities), were conducted to examine differences in DT cognitive performance (based on the accuracy rates) between healthy participants and Parkinson’s patients.

Performance in each task during DT conditions was compared to performance under ST conditions to calculate the interference effects (DT costs; DTCs). A negative sign is applied because higher scores represent poorer performance (e.g., longer times in the ST and DT). As a result, a negative DTC value signifies a decline in performance compared to the ST condition [27]. Therefore, the motor and cognitive DTCs are calculated as follows: Interference effects = −((Performance in DT − Performance in ST)/Performance in ST)*100. After calculating interference effects, a 3 (Group: PIGD, Tremor, Control) × 3 (Condition: DT_SerialSubtraction, DT_Stroop, DT_Clock) × 2 (Domain: Cognitive, Motor) repeated-measures ANCOVA, controlling for weekly exercise-related physical activity (time/week) spent in all exercise-related activities), was conducted. This analysis examined differences in interference patterns between healthy individuals and PD patients, as well as among motor subtypes within the PD group.

Four moderation models (using the PROCESS macro in SPSS [28] with the simple moderation model #1) were employed to assess the moderating effect of weekly exercise-related physical activity (time per week spent in all exercise-related activities) on the relationship between groups (PD-PIGD, PD-Tremor, and Control) and motor performance (time required for the walking task) across the four conditions: (1) ST_Walking, (2) DT_Subtraction, (3) DT_Stroop, and (4) DT_Clock.

#### Calculating Statistical Power

The required sample size for the planned 3 × 4 and 3 × 3 ANCOVA and moderator models was determined using GPower [29]. The input parameters for the a priori power analysis of the ANCOVAs included a medium effect size (f = 0.25), based on conventions for the behavioral sciences [30], a significance level (α) of 0.05, and a power of 0.80. The factor “groups” consisted of three levels, resulting in three groups being analyzed. We have one covariate in the model. The a priori power analysis for the 3 × 4 ANCOVA indicated a required sample size of *n* = 225, and the 3 × 3 ANCOVA indicated a required sample size of *n* = 196 to detect the specified interaction effect with sufficient power.

A power analysis (Superpower; [31]) was conducted for a 3 × 3 × 2 ANCOVA design with repeated measures involving 10 participants per cell (a total of 18 cells in our design). Assuming a medium effect size of f = 0.2828 (according to [32]), an effect of d = 0.4 was considered of interest due to its practical relevance. The value of f is derived from (0.4/√2), with a standard deviation of 1.0 and a correlation of 0.2 (an effect size of d = 0.4 corresponds to a correlation of r = 0.2; [32]). With an alpha level of 0.05, the power analysis showed a power of 100% for the main effect of group, 100% for the main effect of condition, 51.8% for the main effect of angle, 4.0% for the interaction effect of group × condition, 4.8% for the interaction effect of group × domain, 5.1% for condition × domain, and 4.7% for the three-way interaction of group × condition × domain.

GPower was also used to determine the sample size for moderation analysis. Using “Linear multiple regression, fixed model, R^2^ increase, a-priori”, with a medium effect size f of 0.15, a power of 0.80, and an alpha error probability of 0.05 indicated a required sample size of *n* = 55 to detect a moderator effect with sufficient power.

## 3. Results

### 3.1. Participants

There were notable differences in sex distribution between the groups, with the PD-TD group having a higher proportion of men compared to the control group. No significant differences emerged in age, BMI, physical activity, or cognitive function as measured by the Montreal Cognitive Assessment (MoCA; [33]) score. However, there was a significant difference in the Activities Specific Balance Confidence (ABC; [34]) scale, indicating reduced balance confidence in the PD groups, particularly in PD-PIGD compared to controls. The UPDRS-III scores for motor impairment and falls within the last year showed differences between PD subtypes but were not statistically significant (see Table 1).

### 3.2. Effects of Group and Condition on ST and DT Performance

**Figure 1:**
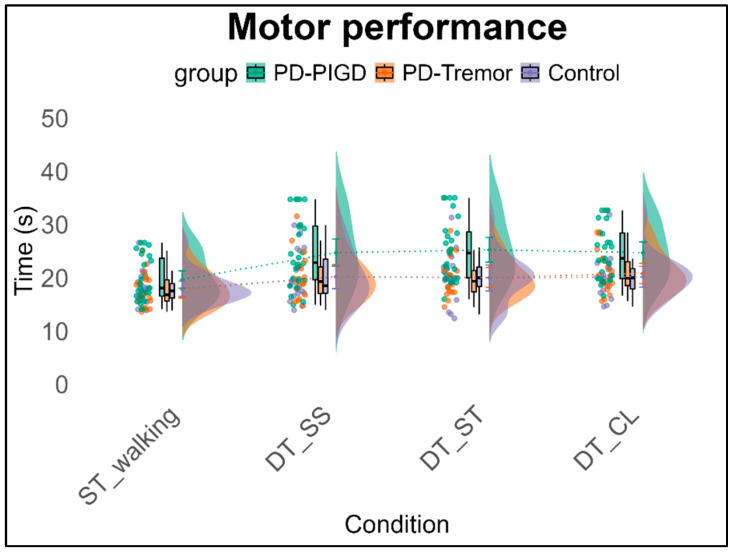
Motor performance (time in s) across groups under single-task and the three dual-task conditions. Note: ST = Single task, DT = Dual task, walking condition, SS = Serial Subtraction task, ST = Stroop task, CL = Clock task.

The 3 × 4 ANCOVA with repeated measures, with walking performance as the within-subject factor, shows a significant main effect of condition, *F*(3,171) = 20.3, *p* < 0.001, *η*^2^_p_ = 0.263; a significant main effect of group, *F*(2,57) = 4.49, *p* < 0.05, *η*^2^_p_ = 0.136; and a significant effect of the covariate for weekly physical activity (time/week spent in all exercise-related activities), *F*(1,57) = 4.81, *p* < 0.05, *η*^2^_p_ = 0.078. Additionally, a significant interaction effect of condition × group is observed, *F*(6,171) = 2.86, *p* < 0.05, *η*^2^_p_ = 0.091, along with a significant interaction effect of condition × covariate, *F*(3,171) = 3.61, *p* < 0.05, *η*^2^_p_ = 0.060. The ST condition differs significantly from all DT conditions (*p* = 0.001), with no significant differences among the DT conditions themselves. A significant difference between groups is observed between the PD-PIGD group and the control group (*p* = 0.026), while the difference between the two Parkinson’s groups is only marginally significant (*p* = 0.08). The interaction effect of condition x group indicates no group differences under the ST condition (walking only). However, in almost all DT conditions, a difference appears between the PIGD and control groups (*p* = 0.01–0.039). Notably, in the Stroop task, the PIGD group differs significantly from both the control group (*p* = 0.01) and the Tremor group (*p* = 0.029) (see Figure 1).

**Figure 2:**
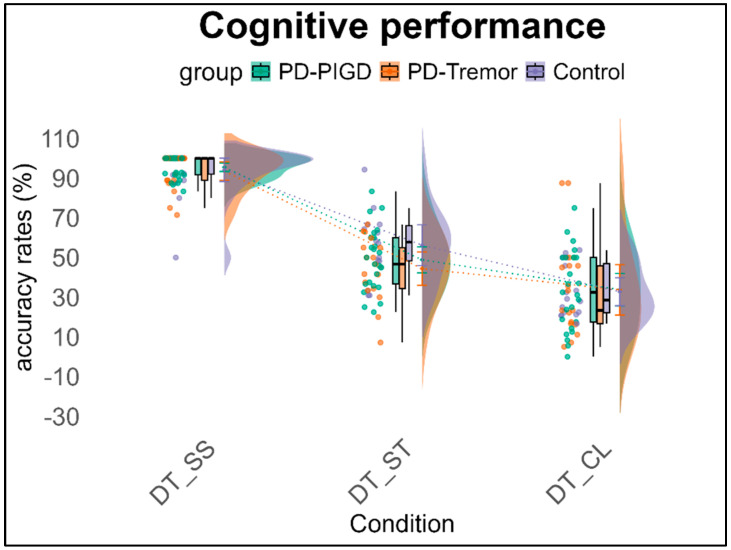
Cognitive performance (accuracy in %) across groups under the three dual-task conditions. Note: DT = Dual task, walking condition, SS = Serial Subtraction task, ST = Stroop task, CL = Clock task.

The 3 × 3 ANOVA with repeated measures on cognitive performance (accuracy rates) shows a significant main effect of condition, *F*(2,98) = 53.5, *p* < 0.001, *η*^2^_p_ = 0.522, with post hoc results indicating that all conditions differ significantly from one another (*p* < 0.001). The main effect of group (*p* = 0.297) and the interaction effect of condition × group (*p* = 0.664) are not significant. The covariate (time per week spent in all exercise-related activities) also has no significant influence (*p* = 0.479) (see Figure 2). The Subtraction task achieves the highest accuracy rate across groups at 95%, followed by the Stroop task, which has a solution rate of 49.9%, close to chance level. The Clock task has the lowest accuracy rate at 32.6%, likely due to unanswered items that, when counted as errors, reduce the solution rate below chance level (see Figure 2).

### 3.3. Interference Effects Across Motor and Cognitive Domains

**Figure 3:**
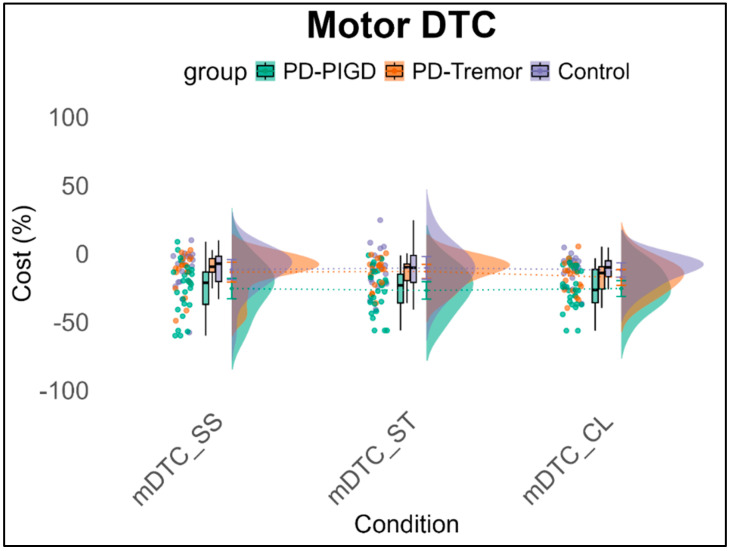
Motor interferences (%) across groups in the dual-task conditions. Note: mDTC = motor dual-task cost, SS = Serial Subtraction task, ST = Stroop task, CL = Clock task.

**Figure 4:**
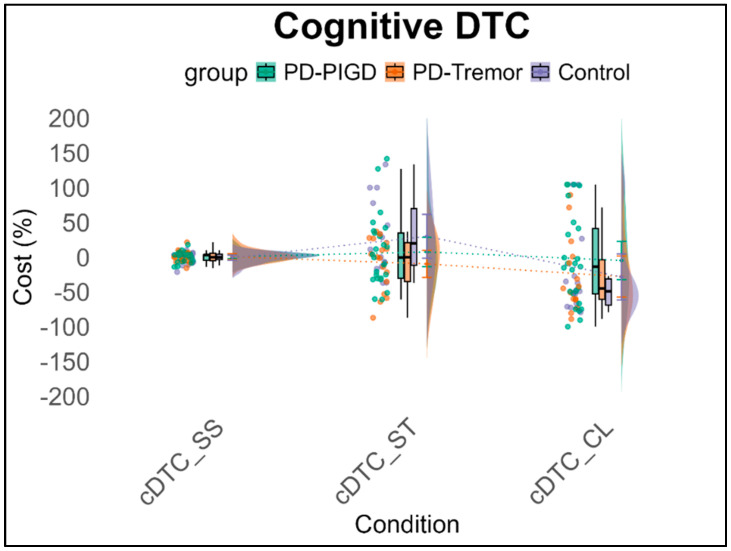
Cognitive interferences (%) across groups in the dual-task conditions. Note: mDTC = motor dual-task cost, SS = Serial Subtraction task, ST = Stroop task, CL = Clock task.

The 3 × 3 × 2 ANCOVA with repeated measures, using motor and cognitive interference as within-subject factors, reveals a significant main effect of domain, *F*(1,90) = 7.99, *p* < 0.01, *η*^2^_p_ = 0.139. On average, a −16.7% interference effect (SE = 1.55) is observed in motor tasks, while −5.96% (SE = 3.74) cognitive impairment is noted. This leads to a mean difference of 12.57% (SE = 4.69) in performance decline between motor and cognitive tasks under dual-task conditions across all conditions. No other significant effects are observed (see Figure 3 and Figure 4).

### 3.4. Direct and Indirect Effects of Group and Physical Activity on DT Walking Performance

The analysis of the four moderator models reveals that, under ST conditions, motor performance is significantly influenced by both group (t = −2.13, *p* = 0.037) and physical activity (t = −2.11, *p* = 0.038). Direct effects of both variables appear in some DT conditions. In the Subtraction task, the group significantly affects motor performance (*t* = −2.76; *p* = 0.008), as does physical activity (*t* = −2.51; *p* = 0.015). In the Stroop task, significant effects of the group (*t* = −2.48; *p* = 0.016) are observed, while physical activity shows only a tendentially significant effect (*t* = −1.86; *p* = 0.07). In the Clock task, the group effect is significant (*t* = −2.09; *p* = 0.041), but physical activity has no significant effect (*t* = −1.16; *p* = 0.251).

The results suggest that time spent in exercise activities does not exert a moderating indirect effect on motor DT performance, irrespective of the cognitive task difficulty. For the ST walking condition, the regression coefficient *β* is −0.083 (*t* = 1.53; *p* = 0.13), showing no significant effect; for the DT walking condition with the Subtraction task, the coefficient is −0.135 (*t* = 1.69; *p* = 0.09); for the Stroop task, the coefficient is 0.073 (*t* = 0.928; *p* = 0.357); and for the Clock task, the coefficient is 0.037 (*t* = 0.504; *p* = 0.616). Even when analyzing the results separately by sex, no indirect effects can be observed. Thus, the results show that the moderator (time/week spent in all exercise-related activities) has no indirect influence on the relationship between the group and the motor DT performance.

## 4. Discussion

This study aimed to comprehensively understand how motor impairments during walking are impacted under ST and DT conditions in patients with PD and healthy controls. The findings highlight several important distinctions in motor–cognitive interference effects across PD motor subtypes, particularly under DT conditions.

A significant difference in motor–cognitive interference was observed between PD patients and healthy controls, especially within the PIGD subtype during DT walking tasks. Notably, the PIGD group displayed greater motor interference effects under DT conditions, particularly during tasks with high cognitive load, such as the Stroop task, compared to the control and TD groups. This finding aligns with previous research suggesting that postural instability and gait difficulty exacerbate cognitive load in motor–cognitive tasks [8] due to basal ganglia dysfunction [11]. Interestingly, while interference patterns differed between PD subtypes, the TD group performance was closer to that of the control group, supporting previous literature that suggests tremor-dominant PD is less associated with gait impairments [9]. Cognitive interference manifests differently in PD motor subtypes. In TD, increased cognitive load tends to exacerbate tremors, which are clinically used to indicate true progression or worsening of the condition. In contrast, for the PIGD subtype, cognitive interference directly impacts gait performance. This distinction is particularly relevant as impaired gait under high cognitive load could serve as a potential biomarker for predicting more severe impairments, including the onset of freezing of gait (FOG). This highlights the importance of tailoring assessment and therapeutic interventions to the specific motor subtype, as cognitive interference’s mechanisms and clinical implications vary substantially between TD and PIGD presentations. Our study underscores the potential value of using DT assessments to differentiate motor subtypes in PD [7], as ST conditions alone did not reveal significant group differences. This distinction could have clinical implications, as DT tasks might provide a more sensitive measure of gait impairment and cognitive load management in PD [35], especially for identifying patients with more severe gait difficulties.

Exercise-related physical activity levels were examined to assess their potential direct moderating effect on motor–cognitive interference. Although regular physical activity has been linked to improved motor function and cognitive resilience in PD [14,36], our findings did not reveal a significant moderating effect of weekly exercise-related physical activity on motor performance under ST or DT. This finding contrasts with prior research suggesting physical activity’s role in reducing symptom progression and preserving gait stability [16]. It is possible that the exercise-related physical activity levels in this sample were insufficient to mitigate the motor–cognitive interference effects of dual-tasking, especially in the PIGD group. On the other hand, our study demonstrates a direct impact of physical activity on motor–cognitive dual-task performance, aligning with findings from studies such as the one by Lauzé and colleagues, which indicate that physical activity can influence PD symptoms [15]. However, this effect tends to be moderate and highly variable. Future research could benefit from investigating the effects of structured, DT-specific interventions to understand better how they impact motor–cognitive interference in different subtypes of PD (see also [37]).

The study has several limitations. First, physical activity was assessed subjectively, making it challenging to quantify activity levels accurately. Objective measurements, such as wearable devices, could provide more precise data in future research. Second, the sample size was relatively small for detecting interaction effects, although it was sufficient for identifying main effects. A larger sample size would enhance the statistical power to explore more complex relationships. Third, motor performance was evaluated solely based on walking times. Incorporating more detailed gait parameters, such as stride length, variability, and stability, could offer a more comprehensive understanding of motor abilities. Lastly, the study’s cross-sectional design limits the ability to infer causal relationships, highlighting the need for longitudinal studies to examine the long-term effects of exercise-related physical activities.

## 5. Conclusions

This study provides valuable insights into motor–cognitive interference effects in PD, particularly under DT conditions, and highlights key differences between motor subtypes. The findings emphasize the heightened motor interference in the PD-PIGD subtype compared to the PD-TD subtype and healthy controls, especially under high cognitive load. These results underscore the utility of DT assessments for distinguishing PD motor subtypes and assessing functional impairments that are not apparent under ST conditions. While physical activity was shown to impact motor performance directly during DT walking, it did not significantly moderate the motor interference effects. This suggests that current activity levels may not be sufficient to counteract motor–cognitive interference. These findings support previous research showing that exercise affects motor and cognitive functions in PD, though its impact is often moderate and varies widely.

Future studies should explore the potential of structured, DT-specific exercise interventions to mitigate motor–cognitive interference, particularly in PD subtypes with pronounced gait (PIGD subtype). Additionally, employing objective measures of exercise-related physical activity and detailed gait parameters, alongside longitudinal designs, could provide a more nuanced understanding of the interplay between physical activity, cognitive load, and motor ST or DT performance in PD. Addressing these gaps could inform the development of targeted, subtype-specific therapeutic strategies, mainly focusing on exercise-related activities, to improve functional independence in individuals with PD.

## Figures and Tables

**Table 1:** Characteristics of PD patients differentiated into motor subtypes PD-TD and PD-PIGD, including mean values (standard deviation) and test values of UPDRS-III.

	Control	PD-TD Ratio ≤ 1.5	PD-PIGD Ratio ≥ 1.0	Stat. Analyses
	(*n* = 19)	(*n* = 18)	(*n* = 27)	
Sex	7 men, 12 women	13 men, 5 women	22 men, 5 women	*CHI*^2^(2) = 10.33, *p* < 0.01, *η*^2^_p_ = 0.402
Age (years)	72.6 (5.53)	67.9 (9.6)	70.2 (7.82)	*F*(2,63) = 1.68, *p* = 0.196, *η*^2^_p_ = 0.052
BMI (kg/m^2^)	27.2 (4.01)	24.8 (4.85)	26.98 (4.28)	*F*(2,60) = 1.76, *p* = 0.181, *η*^2^_p_ = 0.057
UPDRS-III (Score; max = 108)	-	22.8 (6.94)	23.2 (8.15)	*t*(43) = 0.170, *p* = 0.433, *d* = 0.052
Duration of the disease (years)	-	6.22 (4.57)	4.93 (4.31)	*t*(43) = 0.964, *p* = 0.170, *d* = −0.293
ABC-Scale (%)	95.45 (3.75)	89.5 (8.17)	79.5 (19.5) ^ŧ^	*F*(2,62) = 8.16, *p* < 0.001, *η*^2^_p_ = 0.214
Falls last year (*n*; *n* in %, *n* falls)	2 persons (10.5%); 2 falls	5 persons (27.8%); 9 falls	10 persons (37%); 35 falls	*F*(2,14) = 3.91, *p* = 0.951, *η*^2^_p_ = 0.288
MoCA	27.8 (1.44)	27.1 (3.08)	27.5 (1.95)	*F*(2,63) = 0.515, *p* = 0.60, *η*^2^_p_ = 0.028
Physical activity (time/week)	16.1 (9.18)	18.6 (10.4)	12.9 (10.8)	*F*(2,60) = 1.722, *p* = 0.19, *η*^2^_p_ = 0.054

^ŧ^ Significant difference to the control group (*p* < 0.05). The table includes mean values with standard deviations and provides statistical analyses for each variable. Physical activity is assessed by considering the exercise-related activities reported in the CHAMPS questionnaire. ABC = Activities Specific Balance Confidence, MoCA = Montreal Cognitive Assessment, UPDRS-III = Unified Parkinson’s Disease Rating Scale Part III, BMI = Body Mass Index, PD-TD = Parkinson Tremor-Dominant, PD-PIGD = Parkinson Postural Instability and Gait Difficulty.

## Data Availability

Data can be obtained from the corresponding author upon reasonable request. Data are not publicly available due to data protection and ethical restrictions that currently do not allow public disclosure.

## Supplementary Materials

The following supporting information can be downloaded at: https://www.mdpi.com/article/10.3390/brainsci15020114/s1, Inclusion and exclusion criteria for the control group, PD patients with TD, and PD patients with PIGD.
