# Supplementary material for: Exploring Motor–Cognitive Interference Effects and the Influence of Self-Reported Physical Activity on Dual-Task Walking in Parkinson’s Disease and Healthy Older Adults
Source: Brain Sci. 2025 Jan 25;15(2):114. doi: 10.3390/brainsci15020114 (PMC11853502; doi:10.3390/brainsci15020114)
Supplement: Supplementary file 1 [file brainsci-15-00114-s001.zip › brainsci-3412464-supplementary.pdf]

Supplementary Materials

Inclusion and exclusion criteria for the control group, PD patients with TD, and PD patients with PIGD.

	Control	TD	PIGD
Inclusion criteria	- Either gender<br>- Age 50 to 80 years of age<br>- Ability to walk for 10 min continuously unassisted<br>- Able to understand English instructions<br>- Normal or corrected vision	- Either gender<br>- Age 50 to 80 years of age<br>- Diagnosed with idiopathic PD by a Neurologist<br>- Ratio of mean tremor score/mean PIGD score (Jankovic-based classification) is 1.5 or more (with the use of UPDRS-II)<br>- Ability to walk for 10 min continuously unassisted<br>- Able to understand English instructions<br>- Normal or corrected vision	- Either gender<br>- Age 50 to 80 years of age<br>- Diagnosed with idiopathic PD by a Neurologist<br>- Ratio of mean tremor score/mean PIGD score (Jankovic-based classification) is less than or equal to 1.0 (with the use of UPDRS-II)<br>- Ability to walk for 10 min continuously unassisted<br>- Able to understand English instructions<br>- Normal or corrected vision
Exclusion criteria	- A neurological disease other than PD<br>- Had brain surgery in the past, including implanted deep-brain-stimulation	- Tremor score <4 or PIGD score >3 (with the use of UPDRS-II)<br>- A neurological disease other than PD<br>- Had brain surgery in the past, including implanted deep-brain-stimulation	- Tremor score >3 or PIGD score <4 (with the use of UPDRS-II)<br>- A neurological disease other than PD<br>- Had brain surgery in the past, including implanted deep-brain-stimulation

	Control	TD	PIGD
	- Has significant co-morbidities likely to affect gait, e.g., history of stroke<br>- Peripheral neuropathy<br>- Visual impairments that cannot be corrected<br>- Clinically diagnosed with dementia (as stated in the patient's information chart from the patient database at the Sun Life Financial Movement Disorders Research and Rehabilitation)<br>- Is not able to comply with the protocol	- Has significant co-morbidities likely to affect gait, e.g., history of stroke<br>- Peripheral neuropathy<br>- Visual impairments that cannot be corrected<br>- Is unable to walk in the OFF state<br>- Clinically diagnosed with dementia (as stated in the patient's information chart from the patient database at the Sun Life Financial Movement Disorders Research and Rehabilitation)<br>- Is not able to comply with the protocol	- Has significant co-morbidities likely to affect gait, e.g., history of stroke<br>- Peripheral neuropathy<br>- Visual impairments that cannot be corrected<br>- Is unable to walk in the OFF state<br>- Clinically diagnosed with dementia (as stated in the patient's information chart from the patient database at the Sun Life Financial Movement Disorders Research and Rehabilitation)<br>- Is not able to comply with the protocol
